# T-cell immunity against senescence: potential role and perspectives

**DOI:** 10.3389/fimmu.2024.1360109

**Published:** 2024-03-05

**Authors:** Kseniia Matveeva, Mariia Vasilieva, Ekaterina Minskaia, Stanislav Rybtsov, Daniil Shevyrev

**Affiliations:** Sirius University of Science and Technology, Sirius, Russia

**Keywords:** senescence, T-cells (or lymphocytes), adaptive immunity, SASP (senescence-associated secretory phenotype), retroelement, transposable element (TE), senolytic agent, immunoaging

## Abstract

The development of age-associated diseases is related to the accumulation of senescent cells in the body. These are old non-functional cells with impaired metabolism, which are unable to divide. Such cells are also resistant to programmed cell death and prone to spontaneous production of some inflammatory factors. The accumulation of senescent cells is related to the age-associated dysfunction of organs and tissues as well as chronic inflammation that enhances with age. In the young organism, senescent cells are removed with the innate immunity system. However, the efficiency of this process decreases with age. Nowadays, more and more evidences are accumulating to support the involvement of specific immunity and T-lymphocytes in the fight against senescent cells. It has great physiological importance since the efficient elimination of senescent cells requires a high diversity of antigen-recognizing receptors to cover the entire spectrum of senescent-associated antigens with high precision and specificity. Developing the approaches of T-cell immunity stimulation to generate or amplify a physiological immune response against senescent cells can provide new perspectives to extend active longevity. In this mini-review, the authors summarize the current understanding of the role of T-cell immunity in the fight against senescent cells and discuss the prospects of stimulating adaptive immunity for combating the accumulation of senescent cells that occurs with age.

## Introduction

Due to scientific, technological, and medical progress, there is a significant increase in life expectancy. According to global statistics, over the last century and a half life expectancy increased from ~30 to ~73.5 years (1–3). This led to a significant growth in the proportion of elderly people and to demographic aging in most modern societies. According to the UN data from 2010, the world population over 60 years was about 800 million and it is projected to exceed 2 billion by 2050, with the proportion of the elderly population increasing from 9.8% in 2022 to 22% by 2050 (4, 5).

Elderly age is associated with an increased risk of cardiovascular, cancerous, and autoimmune diseases. This also takes place in reduced overall body resistance, as well as severe and prolonged infectious diseases (6–11). Therefore, the increasing proportion of the elderly population places a heavy socioeconomic burden on the budgets of developed and developing countries and seriously affects their healthcare systems (12). This leads to a forced rise in the retirement age and makes the extension of the active longevity period one of the most serious social challenges of our time.

The aging at the tissue level is associated with the formation and accumulation of senescent cells (13, 14). Such cells are characterized by resistance to apoptosis, irreversible cell cycle arrest, mitochondrial dysfunction, a specific secretory phenotype (SASP), and abnormalities in the protein quality control machinery (15). Various DNA damage, replicative depletion, oxidative stress, and activation of tumor suppressor genes can induce cell senescence (16). The proliferation arrest in senescent cells prevents their malignization, and the secretion of SASP factors can promote tissue repair and remodeling which is especially observed during embryogenesis and the early postnatal period (17, 18). However, the beneficial effects of senescent cells are replaced by harmful ones in old age (18, 19). In a young organism, normal tissue homeostasis is maintained by the appropriate removal of senescent cells, but the efficiency of this process decreases with age progression (18–20). Thus, the accumulation of senescent cells in aging is associated with an increased risk of cardiovascular, autoimmune, and cancerous diseases, as well as high susceptibility to various infections (21–28). In addition, it reduces the efficiency of repair processes (29). It was shown that the accumulation of such cells accelerates the development of many diseases, including atherosclerosis, osteoporosis, osteoarthritis, Parkinson’s disease, Alzheimer’s disease, etc. (21–29).

The immune system plays a major role in the elimination of senescent cells by responding to the SASP phenotype (30, 31). Its components include growth factors, chemokines and cytokines, proteases, lipids, and extracellular vesicles (32, 33). Many of these factors are pro-inflammatory and attract macrophages, NK and NKT cells, neutrophils, other immune system cells, and natural IgM antibodies that provide the removal of old cells (30, 31, 34). On the other side, immune cells are also susceptible to aging. Thus, with age, the immune system loses its ability to remove senescent cells from tissues promptly, and the vicious circle gradually closes (34).

In recent years, there has been increasing attention to the development of methods that allow the selective removal of old cells. New classes of drugs are being developed that kill senescent cells (senolytics) or reduce the negative impact of SASP inflammatory factors (senomorphics) (35, 36). In parallel, attempts are being made to adapt CAR-T technologies to target senescent cells (37). Potentially, it would be possible to maintain the homeostasis of aging tissues and slow down the aging process. For example, encouraging results were obtained in transgenic mice in which the elimination of senescent cells reduced age-related organ dysfunction and prolonged the life span (38). However, some limitations prevent the establishment of a safe and effective therapeutic approach to target senescent cells. Cell surface markers of senescence, which were specified above, are not senescence-specific and may be present in normal cells. Moreover, their expression levels differ between tissues which prevents their safe use as therapeutic targets. Many existing senolytics target specific signaling pathways that are activated during cellular aging, such as mTOR and cGAS (32, 36). However, the existence of an intracellular signaling factor unique to senescent cells is controversial due to the complex organization and pleiotropy of the intracellular signaling machinery. This reduces the selectivity of action against senescent cells (33, 39). From this side, exploiting the metabolic features of senescent cells can provide more interesting results. It is known that senescent cells are characterized by increased activity of lysosomal β-galactosidase in the shifted pH optimum (~6.0). Positive results were obtained in experiments using a prodrug, in which active metabolite is formed in the lysosomes of senescent cells and causes their death. The use of such therapeutics led to the recovery of physiological functions and reduced the signs of systemic inflammation in elderly mice. Moreover, its action was relatively selective and no significant negative effects on normal cells were detected (40). However, increased β-galactosidase activity at pH~6.0 was observed in some normal tissues, such as neurons and retinal cells, and the activity of this enzyme in healthy cells may increase during the some metabolic process (41–43). Therefore, this approach also has significant limitations.

Thus, despite the encouraging results of recent years in the field of anti-aging therapies (36), the problem of finding and developing an effective and safe method for the elimination of senescent cells with the potential for translation into clinical medicine is still acute.

## Fight senescence: the potential role of T-cell immunity

It is well known that innate immunity is responsible for the elimination of senescent cells (44). However, the role of the adaptive immune system in this process is poorly understood (30). It was shown that senescent cells in different tissues can upregulate the expression of classical and non-classical MHC-I molecules (45) and in some cases MHC-II molecules (46). Some studies support the role of CD4^+^ and CD8^+^ lymphocytes in the destruction of senescent cells (30). However, it should be noted that increased expression of non-classical HLA-E molecules protects senescent cells by suppressing the activity of NK and CD8^+^ lymphocytes (47). Possibly, this is one of the mechanisms preventing the removal of senescent cells from tissues in old age.

**Figure 1:**
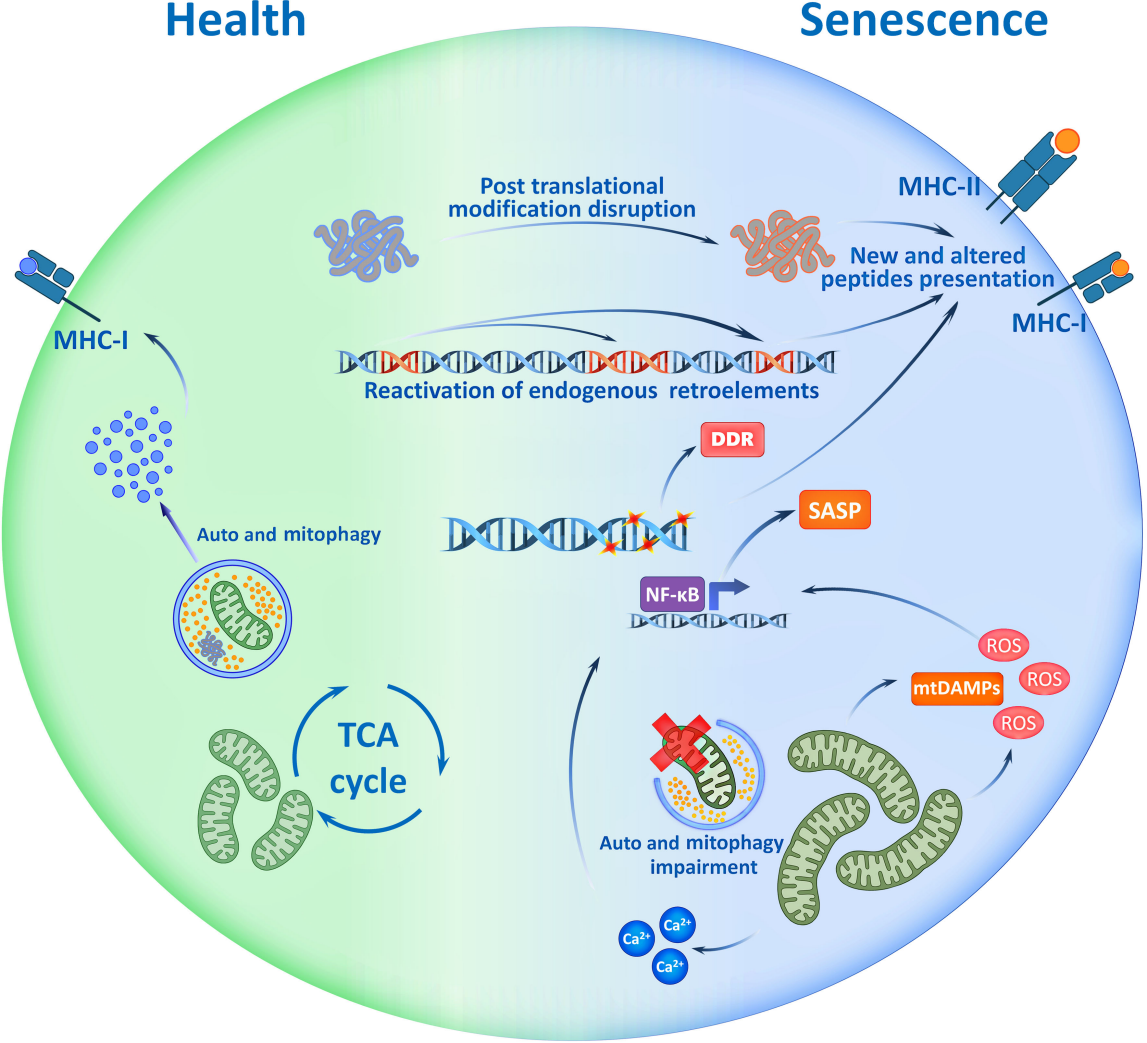
Potential Immunogenicity of Senescent cell. Accumulation of mutations, disruption of post translational modifications of proteins, activation of mobile retroelements and signs of genome instability - all are the traits of senescent cells which may be reflected in immunopeptidome. Cell ageing accompanies with mitochondrial dysfunction that causes mtDAMP release, ROS production and increase of Ca^2+^ ions. These factors drive the SASP, and with the immunopeptidome changes, shape the immunogenicity of senescent cells. SASP, Senescence Associated Secretory Phenotype; TCA, Tricarboxylic Acid; ROS, Reactive Oxygen Species; mtDAMP, mitochondrial Danger Associated Molecular Patterns; DDR, DNA Damage Response; MHC, Major Histocompatibility Complex.

In recent studies, it was shown that senescent cells demonstrate changes in their proteome and, consequently, their immunopeptidome (**Figure 1**). Thus, senescent cells present aging-associated antigens – approximately 10% of all epitopes in complex with MHC-I are unique to senescent cells and cannot be found in non-senescent analogs (45). Recent studies using *in vivo* models demonstrated the ability of senescent antigens to trigger a strong CD8^+^-lymphocyte response, which challenges the notion of low immunogenicity of such epitopes (45, 48). Thus, due to the production of SASP and DAMP (damage-associated molecular patterns) factors, senescent cells demonstrate adjuvant properties – they effectively recruit dendritic cells, cause their activation, and stimulate their maturation (49, 50). It is also known that some types of senescent cells enhance the expression of MHC-II molecules which helps directly present antigens to CD4^+^-lymphocytes without the participation of professional antigen-presenting cells (45, 46). All this triggers an adaptive immune response to combat senescent cells.

In the context of central tolerance, the presence of T-lymphocytes that specifically recognize senescence-associated epitopes does not always seem obvious, but the changes associated with cell aging should be taken into account. For example, the accumulation of mutations leads to changes in the antigenic structure of various proteins (51, 52). Disruption of the processes of post-translational modifications of proteins, which is characteristic of senescent cells, also affects the antigenic properties of epitopes presented in MHC molecules (53). In addition, genome instability increases with age and cells undergo activation of transposable elements and formation of particles similar to retroviruses, which makes a serious contribution to aging and accompanies the transition of cells to the senescent state (54). At the same time, aging is associated with the activation of non-LTR elements (without Long Terminal Repeats) which are evolutionarily younger, while in the thymus there is mainly a presentation of antigens of LTR elements (54, 55). In other words, central tolerance practically cannot be formed to antigens of aging-associated mobile elements. A recent study shows that spontaneous commensal cytomegalovirus (HCMV) reactivation in senescent fibroblasts and presentation of glycoprotein-B epitopes of HCMV in MHC-II complexes makes cytotoxic CD4^+^ lymphocytes eliminate senescent skin fibroblasts in an MHC-II-dependent manner (56, 57). This is consistent with the ideas about the activation of genomic mobile elements and reactivation of commensal viruses in senescent cells and the important role of these processes in the elimination of senescent cells by the mechanisms of adaptive immunity. Taking into account these features, it becomes obvious that the immunopeptidome of senescent cells has a high immunogenic potential, which can help to develop personalized therapeutic approaches to prevent or mitigate the negative effects of aging.

## Perspectives

Recent studies focused mainly on the role of senescence in tumor physiology (58–61). It is currently believed that the transition of tumor cells to the senescent state plays a protective role and inhibits their proliferation due to cell cycle arrest and an increase in their immunogenicity (45, 62, 63). The last one seems to be related to the production of SASP and DAMP factors,high expression of MHC molecules, alteration of the proteome, and enhanced presentation of senescence-associated autoantigens that can activate T-lymphocytes (45, 46, 50, 64). Many anticancer therapies have been shown to induce tumor cell senescence, which promotes the recruitment and activation of CD4^+^ and CD8^+^ lymphocytes and enhances antitumor protection (64, 65). Interestingly, immunization with derivatives of senescent tumor cells induces a stronger and more effective antitumor immune response than immunization with derivatives of tumor cells after immune-mediated tumor cell death (48, 63, 66). In other words, induction of senescence makes it possible to solve the problem of low immunogenicity of autologous tumor antigens. The reason for that is probably the secretion of SASP factors, especially IFNγ and TNFα, as well as the reactivation of commensal viruses and genomic non-LTRs that alter the immunopeptidome of senescent cells (54, 56, 57, 67–75).

In recent years, various drug delivery systems based on exosomes loaded with target substances have been intensively developed. Thus, relatively simple and cheap methods were designed to produce cell-derived nanovesicles providing high safety, bioavailability, and the possibility of a personalized approach (76–78). For example, a recent study shows that the use of senescent tumor cell nanovesicles as a personalized therapeutic vaccine provides high immunogenicity and induces strong antitumor immunity with no exogenous adjuvants (63). The role of endogenous retrotransposons in cancer initiation and progression is well known in the context of their mutagenic activity. Increased activity of non-LTR elements is found in many tumor types (79–84). However, the formation of an adequate immune response to non-LTR epitopes does not occur due to the inhibitory influence of the tumor microenvironment and the dualistic role of senescent cells in tumors (82, 85). It can be assumed that the use of nanovesicles of tumor cells with induced senescence as a vaccine creates an effective T-cell immune response against epitopes of non-LTR elements since its formation occurs outside the inhibitory tumor microenvironment (48, 56, 57). Such antitumor immune response is probably produced not by tumor neoantigens but by epitopes of retrotransposons whose activity is increased in the tumor. However, we need additional studies for a more detailed understanding of the role of senescence in the enhancement of antitumor immunity, taking into account the high heterogeneity of the tumor and its microenvironment concerning the dynamics of senescence onset.

In the context of high immunogenicity, the use of senescent cell nanovesicles to stimulate an “antisenescent” immune response seems to be a promising and safe method to combat age-related senescent cell accumulation (63, 86, 87). However, there are currently no studies addressing this issue. The decreased efficiency of senescent cell elimination may be related to the depletion of adaptive immunity reserves or the formation of peripheral tolerance to senescent-associated antigens with age (88–91). This is probably facilitated by prolonged and increasing stimulation of various antigens of senescent cells, including antigens of non-LTR elements or commensal viruses, whose activity is enhanced with aging (92–94). Senescent cell nanovesicles are highly immunogenic and contain all potential senescent-associated antigens that result from mutations, disruption of post-translational modifications of proteins, or activation of transposable elements (63, 76–78). This approach has a potential advantage in the context of the formation or amplification of an “antisenescent” immune response. It may help to avoid the complex process of identifying individual sets of specific senescent-associated antigens.

Another promising method of stimulating the “antisenescent” immune response could be the development of personalized polyepitope mRNA vaccines against antigens of non-LTR elements or commensal viruses, which activity is increased in the senescent cells in some individuals (95–98). However, the development of vaccines against herpesviruses, retroviruses, and transposable elements is a complex and nontrivial task that has yet to be solved (99). Another promising senotherapy approach may be selective inhibitors of endogenous LINE-1 reverse transcriptases that do not inhibit the activity of the telomerase complex and mitochondrial DNA polymerases (100, 101). In mouse experiments, reverse transcriptase inhibitor therapy reduces signs of age-related inflammaging by decreasing the number of senescent cells (102, 103). Also, in a recent population-based study, transcriptase inhibitors-based therapy in HIV-infected patients lowered their biological age (101). However, the low selectivity and the possibility of inhibiting the activity of the telomerase complex and mitochondrial DNA polymerases carries a high risk of side effects, which limits the use of such therapeutics in aging therapy (104–106). Similar limitations were observed in most studies of the impact of reverse transcriptase inhibitors on biological age (104–108).

The development of CAR-T technology made it possible to target and harness the power of T-cell immunity against specific antigens, including those associated with senescence. For example, it was found that the level of expression of uPAR (urokinase-type plasminogen activator receptor) and NKG2DLs (natural killer group 2 member D ligands) increases in aging and senescent cells in mice and primates (37, 109, 110). Recent studies demonstrated that elimination of these cells using senolytic CAR-T cells reversed senescence-associated pathologies and ameliorated metabolic dysfunction without adverse effects (37, 109, 110). Senolytic CAR-T cells appear to have prophylactic potential (110). However, their targets (uPAR and NKG2DLs) not only participate in cell aging but also play a physiological role. This could potentially limit the use of such an approach in clinical practice, requiring rigorous investigations to address its safety.

## Conclusion

It is well known that the risk of multiple pathologies increases with age, including autoimmune, cancerous, infectious diseases, and metabolic disorders, while the regenerative potential of various tissues declines. Despite these conditions having different pathogenesis, they are marked by a growing number of aging and senescent cells accumulating with age (111–114). At a young age, such cells are removed by immune mechanisms, but their elimination efficiency decreases with time. The aging of the immune system itself and the formation of peripheral tolerance under prolonged and increasing stimulation by senescence-associated antigens may play a central role in such processes (45, 89, 115–117). Restoration of immunoreactivity against senescent cells will slow down the aging processes and maintain homeostasis and the functions of aging tissues. However, this is a challenging task, and the solution must take into account the safety and selectivity of the approaches (33, 35, 39), despite many of them being currently under development and showing promising results (19, 35–38). Therefore, the development of safe and accurate methods for the removal of senescent cells, taking into accounts their high heterogeneity and antigenic diversity, is currently an acute task. The approaches to intensify or generate an immune response against senescent-associated antigens have great potential in this context due to the adaptive immunity has great precision and specificity of action, as well as a high diversity of antigen-recognizing receptors to cover the entire spectrum of senescence-associated antigens.

## Author contributions

KM: Conceptualization, Writing – original draft. MV: Conceptualization, Supervision, Writing – original draft. EM: Conceptualization, Formal analysis, Supervision, Writing – review & editing. SR: Conceptualization, Formal analysis, Supervision, Writing – review & editing. DS: Conceptualization, Formal analysis, Project administration, Supervision, Visualization, Writing – review & editing, Writing – original draft.
